# Craniosacral therapy for migraine: Protocol development for an exploratory controlled clinical trial

**DOI:** 10.1186/1472-6882-8-28

**Published:** 2008-06-09

**Authors:** John D Mann, Keturah R Faurot, Laurel Wilkinson, Peter Curtis, Remy R Coeytaux, Chirayath Suchindran, Susan A Gaylord

**Affiliations:** 1 Department of Neurology, School of Medicine, University of North Carolina at Chapel Hill, Chapel Hill, NC, USA; 2 Program on Integrative Medicine, Department of Physical Medicine and Rehabilitation, School of Medicine, University of North Carolina at Chapel Hill, Chapel Hill, NC, USA; 3 Integrative Health Center of Chapel Hill, Chapel Hill, NC, USA; 4 Department of Family Medicine, School of Medicine, University of North Carolina at Chapel Hill, Chapel Hill, NC, USA; 5 Department of Biostatistics, School of Public Health, University of North Carolina at Chapel Hill, Chapel Hill, NC, USA

## Abstract

**Background:**

Migraine affects approximately 20% of the population. Conventional care for migraine is suboptimal; overuse of medications for the treatment of episodic migraines is a risk factor for developing chronic daily headache. The study of non-pharmaceutical approaches for prevention of migraine headaches is therefore warranted. Craniosacral therapy (CST) is a popular non-pharmacological approach to the treatment or prevention of migraine headaches for which there is limited evidence of safety and efficacy. In this paper, we describe an ongoing feasibility study to assess the safety and efficacy of CST in the treatment of migraine, using a rigorous and innovative randomized controlled study design involving low-strength static magnets (LSSM) as an attention control intervention.

**Methods:**

The trial is designed to test the hypothesis that, compared to those receiving usual care plus a treatment with low-strength static magnets (attention-control complementary therapy), subjects receiving usual medical care plus CST will demonstrate significant improvement in: quality-of-life as measured by the Headache Impact Test (HIT-6); reduced frequency of migraine; and a perception of clinical benefit. Criteria for inclusion are either gender, age > 11, English or Spanish speaking, meeting the International Classification of Headache Disorders (ICHD) criteria for migraine with or without aura, a headache frequency of 5 to 15 per month over at least two years. After an 8 week baseline phase, eligible subjects are randomized to either CST or an attention control intervention, low strength static magnets (LSSM). To evaluate possible therapist bias, videotaped encounters are analyzed to assess for any systematic group differences in interactions with subjects.

**Results:**

169 individuals have been screened for eligibility, of which 109 were eligible for the study. Five did not qualify during the baseline phase because of inadequate headache frequency. Nineteen have withdrawn from the study after giving consent.

**Conclusion:**

This report endorses the feasibility of undertaking a rigorous randomized clinical trial of CST for migraine using a standardized CST protocol and an innovative control protocol developed for the study. Subjects are able and willing to complete detailed headache diaries during an 8-week baseline period, with few dropouts during the study period, indicating the acceptability of both interventions.

**Trial Registration:**

ClinicalTrials.gov NCT00665236

## Background

Migraine affects approximately 20 percent of the adult population, developing in the second and third decades and often persisting into late middle age and beyond [1,2]. Although the pathophysiology is not fully understood, there are clearly genetic, vascular and neural mechanisms involved [3]. Frequency and duration are highly variable and the onset is said to be triggered at times by stress, history of head or neck trauma, certain foods and hormonal changes in women [4-6]. Symptoms can be difficult to control and quality of life may deteriorate substantially.

Management includes modification of diet, and recommendations for sleep, exercise, and stress reduction through bio-behavioral interventions as well as the use of a range of medications for prevention and acute treatment of migraine. Many of these medications are associated with significant side effects [7,8]. Furthermore, overuse of medications for the treatment of episodic migraines is a risk factor for developing chronic daily headache [9]. The study of non-pharmacological interventions for the treatment or prevention of migraine headaches is therefore warranted. Because conventional care for migraine is suboptimal, many individuals seek alternative treatment options [10,11].

Craniosacral therapy (CST) is one popular non-pharmacological approach to the treatment or prevention of migraine headaches for which there is limited evidence of safety and efficacy.

CST involves manually identifying restrictions in the craniosacral system which includes the bones, membranes and cerebrospinal fluid (CSF) that surround the brain and spinal cord, and using soft, gentle hands-on techniques to both normalize the CST fluid rhythm and correct restrictions in perispinal tissues and fascia [12]. Manual palpation and manipulation of this system theoretically affects sensory, motor, cognitive and emotional processes in the nervous system [12-15].

CST is often used as adjunctive therapy by physical therapists, osteopathic physicians, chiropractors and massage therapists. It is purported to reduce the use of conventional pain medications and improve daily functioning in a variety of conditions [16]. To our knowledge, there are no reports in the conventional medical literature of the use of CST for migraine, either alone or in combination with standard medical care. Several reports of CST in general headache suggest the possibility of significant benefit for some patients, including reduction of intensity of pain, reduction in medication use and improved function [16,17]. These reports suffer from a lack of specific headache diagnoses and poor documentation of how CST was integrated with conventional care. Criteria for diagnosis, numbers of treatments, co-morbid conditions, psychosocial status, coping strategies for pain, prior trauma, qualifications of the practitioner, longitudinal follow-up, and changes in quality-of-life and overall function are not reported. Moreover, these study designs lack control groups and randomization.

In this paper, we describe an ongoing feasibility study to assess the safety and efficacy of CST in the treatment of migraine. This is the first rigorous approach to the study of CST for migraine. The aims of the study are as follows:

1) To determine the feasibility of developing a clinical trial comparing usual medical care plus craniosacral therapy (CST) *versus* usual medical care and low-strength static magnet therapy (LSSM – attention-control) as treatments for the prevention of migraine;

2) To identify relevant secondary outcomes associated with usual care plus adjunctive CST; and

3) To identify and find solutions for potential problems in conducting a future large clinical trial to assess the efficacy of CST for the prevention of migraine.

## Methods

### Study Design

**Figure 1:**
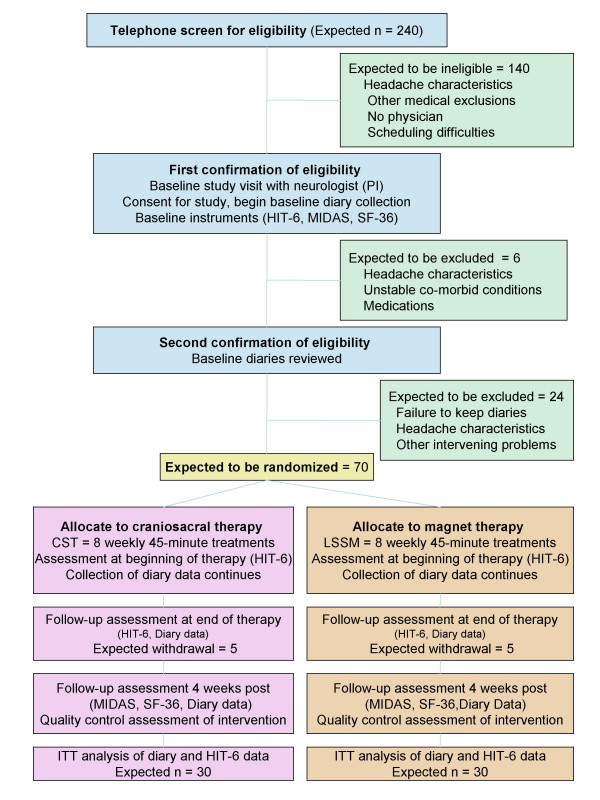
Recruitment, selection and randomization flow diagram for the clinical feasibility trial of craniosacral therapy (CST), 2006–2008

The study examines the feasibility of a randomized controlled trial that compares CST to low strength static magnets (LSSM). Figure 1 illustrates the overall design and subject flow through the study. Study procedures and consent forms were reviewed and approved by the Institutional Review Board of the University of North Carolina.

### Hypothesis

The trial is designed to test the hypothesis that, compared to those receiving usual care plus a treatment with low-strength static magnets (attention-control complementary therapy), subjects receiving usual medical care plus CST will demonstrate significant improvement in: quality-of-life as measured by the Headache Impact Test (HIT-6), a headache-specific quality-of-life measure; reduced frequency of migraine; and a perception of clinical benefit.

### Eligibility

Criteria for inclusion are: individuals of either gender, age 12 and above, English or Spanish speaking, meeting the International Classification of Headache Disorders (ICHD) criteria for migraine with or without aura, a headache frequency of 5 to 15 per month and a history of at least two years of migraine (Table 1). The rationale for headache frequency criteria is that patients with more than 5 headaches per month could be considered to have failed conventional therapy, especially if function is significantly impaired, while those with more than 15 headaches per month are considered to have chronic migraine and may be physiologically different than those with episodic migraine [18]. Children are included because migraine is a significant problem in children, with limitations in pharmacologic approaches [19].

**Table 1:** Eligibility of Subjects for Clinical Feasibility Trial of Craniosacral Therapy, 2006–2008

**Inclusion Criteria**	**Exclusion Criteria**
Subjects > 11 years of age	Marked depression, anxiety or psychosis
Either gender	More than 2 visits/month for mental health care
Meet ICHD* criteria for migraine	More than one psychiatric medication
Headache frequency/month: 5–15	Major medical illness under treatment
Headache history > 2 years	Pregnancy
Willing to complete daily diary	Clotting disorders
Able to attend 8 weekly treatments	Head or neck trauma in past 2 years
	Cranial/neck surgery in past 2 years
	Cardiac Pacemaker
	Other implanted or external electrical device (e.g. insulin pump)

*International Classification of Headache Disorders, defined by expert members of the International Headache Society

Exclusion criteria include marked depression, anxiety or psychosis; more than two visits/month for mental health care; more than one psychiatric medication; major medical illness under treatment; pregnancy; clotting disorders; recent head or neck trauma within two years, and presence of a cardiac pacemaker or other implanted or external electrical device (see Table 1). While there is no evidence that CST or magnet therapy would harm either the mother or the fetus, the frequency of migraine is often affected by hormonal fluxes such as during and following pregnancy [20]. Hence, women were excluded who were pregnant, nursing, or initiating hormone replacement therapy. Those with mental illness requiring multiple medications, and those with diagnoses of major depression, anxiety or psychosis, were excluded because of the requirement of detailed daily diary completion as well as the effects of active mental illness in exacerbating migraine. To increase the believability of the LSSM intervention, those with cardiac pacemaker or other electrical devices were excluded, even though it was unlikely that the extremely low-strength magnets would affect these devices.

### Recruitment

Subjects are recruited from the following sources: 1) University of North Carolina (UNC) Headache Clinic; 2) local neurology practices; 3) local primary care clinics; 4) broadcast email to UNC students and employees; and 5) advertisements in the regional press. Subjects receive eight sessions of CST or magnet therapy at no cost. They are reimbursed for completing their diaries and questionnaires up to a maximum of $170 for the entire study. Recruitment and study materials are available in both Spanish and English. Hispanic subjects are recruited through advertisements in Spanish-language newspapers and through clinics serving the Hispanic community. A Spanish interpreter is available for help with consenting and to accompany subjects at each treatment session.

The recruitment strategy has been intentionally broad, targeting headache specialty care, primary care and non-care populations with migraine to avoid missing qualifying subjects and to include subjects with differing levels of care and a range of migraine symptoms. Although migraine is a common disorder, many sufferers either do not seek medical attention or, when they do, are not treated for migraine [21,22]. Random digit surveys of the general public suggest that less than 50% of those with migraine are currently seeing a physician for that problem [23]. Therefore, limiting recruitment to those under active medical care for migraine would potentially eliminate more than half the eligible individuals from consideration. In addition, only a small subset of migraine patients seek specialty care; these individuals may be fundamentally different from the global population of migraine patients [21]. Limiting recruitment to those individuals would restrict the pool of potential subjects.

### Screening, consent, and enrollment

Those interested in being part of the study contact the research staff for a telephone screening interview. Those identified through the screening process as potentially eligible subjects are scheduled to meet with the study coordinator for the consenting process and to undergo a baseline medical assessment, including a complete medical history and physical examination by the study neurologist. The study neurologist confirms the diagnosis of migraine and excludes from the study those individuals with other medical problems that could put the patient at risk or confound the study results, e.g., recent head trauma, untreated or uncontrolled hypertension, diabetes, or depression. Excluded patients are referred back to their physicians for further medical management. Enrolled subjects are asked to avoid changes in medications, dietary supplements, and non-pharmacologic treatments for headache during the course of the study.

### Baseline Period

Eligible subjects are instructed in the maintenance of a daily headache diary for eight consecutive weeks while continuing usual care. Subjects have the option of completing the diary either on paper and mailing it to the study coordinator, or on a secure internet website. After they have completed 4 weeks of diaries, their entries are reviewed with the study neurologist to determine eligibility for the treatment phase of the study. Eligibility consists of completion of at least 80% of headache diaries and report of at least 5 migraines. Eligible subjects are called and scheduled to visit the therapist for eight weekly sessions. They are asked to continue their headache diaries for the full 20-week duration of the study.

### Randomization

Subjects are randomized to one of the two treatment arms using a concealed allocation protocol. At the subject's first treatment visit, the study therapist performs a brief intake interview and then opens an opaque, sequentially numbered envelope that identifies which treatment group has been randomly selected for this subject. The randomization scheme uses a permuted block method to generate the intervention assignment sequence being implemented at the treatment location to assure that the randomization balance is maintained throughout enrollment [24].

### Study therapist

The study therapist, Laurel J. Wilkinson, is a NC licensed massage and body therapist and registered nurse. She trained in CST under John E. Upledger, M.D. in Michigan in 1979–1980 and then completed advanced CST training at the Upledger Institute *[Upledger Institute, 11211 Prosperity Farms # D325, Palm Beach Gardens, FL 33410]* from 1986–1989. Ms. Wilkinson has utilized CST full-time in her practice for the past 18 years. She is an approved Upledger Institute Instructor for CST classes.

We chose to use only one therapist because, with training and close monitoring, we theorized that a single experienced individual was more likely to achieve a standardized approach to each subject than several therapists in a modality where only weak reliability between practitioners in diagnosis and palpation techniques has been reported [25-27]. Also, there is evidence that using more than one CST therapist in a study can dilute the outcome effect [28].

To evaluate possible therapist bias, half of all encounters with subjects are videotaped. Selected recordings are analyzed qualitatively to assess for any systematic differences in her interactions with subjects in the two groups.

### Masking

Because of the nature of the interventions, the therapist and subjects cannot be masked. In addition, those individuals on the study team who review the video tapes are unmasked. Other study personnel may learn of treatment assignment in the course of reviewing diaries or other data. Because the study neurologist conducts post treatment exit interviews with the subjects, his masking is broken at that time. Only the biostatistician is completely unaware of the treatment assignment.

### The Intervention Group – CST

Subjects in the intervention group receive usual medical care plus one CST treatment per week for 8 weeks. Because no studies have reported usual or evidence-based treatment schedules for CST, the number of treatments was based on data from our initial, unpublished CST practitioner survey and opinions of experienced therapists in the field. The subject lies supine and fully clothed, except for belts, watches, and shoes, while the therapist proceeds with an evaluation of the craniosacral system. She then assesses and treats connective tissue restrictions of the lower and upper body, the neck, all of the cranial bones and underlying tissues.

The CST protocol for each subject follows the Upledger Institute approach using the elements shown in Table 2. The therapist keeps detailed notes on an assessment and treatment form developed for the study. Because CST may vary among individual migraine patients based on the therapist's assessment, complete standardization of the treatment protocol was not possible. The therapist may elect to work on any fascial restrictions she finds, as in an ankle or shoulder. At each visit, the protocol calls for a symptom history covering the previous week, blood pressure and heart rate determinations before and after treatment, and a summative global assessment of the craniosacral system (CSF rhythm and points of restricted movements).

**Table 2:** Summary of procedural sequence at each visit for craniosacral therapy (CST), 2007–2008

1.	Brief review of recent headache symptoms and general symptoms and assessment of any adverse effects of treatment
2.	Evaluation of the craniosacral rhythm*, including amplitude, quality, and rate
3.	Arcing, palpating for active lesions – checking for fascial restrictions^†^
4.	Fascia releases at pelvis, dural tube traction, L5-S1 decompression, sacro-iliac decompression^§^
5.	Fascia releases at lower respiratory, thoracic inlet, hyoid, and cranial base
6.	Vertical membrane (falx cerebri) system evaluation and treatment using frontal and parietal bone and soft tissue manipulations
7.	Horizontal membrane system (tentorium) evaluation and treatment using sphenoid and temporal bone and soft tissue manipulations
8.	Mandibular compression and decompression
9.	Hard palate intra-oral evaluation and treatment
	a. illary-palatine: flexion/extension, torsion, shear, compression
	b. vomer evaluation: flexion/extension, torsion, shear, compression
	c. palatine
	d. zygoma and nasal bones
10.	Dural tube evaluation – mobility, tension and restrictions^#^
11.	Still-point induction**
12.	Global assessment with percentage improvement

* The craniosacral rhythm is palpated by the practitioner (at the feet) and assessed for rate, quality, and symmetry. A normal rate is 8–12 cycles per minutes. A normal rhythm shows a balanced vitality.

† Arcing is a gentle traction technique for detecting fascial restrictions.

§ Decompression allows for the release of joint and soft-tissue restrictions.

# Dural tube, the sheath of connective tissue surrounding the spinal cord, is evaluated through manual palpation of the spine between the occiput and the sacrum.

** Still point induction is a manual technique performed at the occiput, designed to bring the craniosacral rhythm to a therapeutic pause.

### The Attention Control Group – Low strength static magnets (LSSM)

We selected low strength static magnet (LSSM) therapy as the control intervention on the basis that it was a recognized and popular alternative treatment for pain syndromes, had a valid biological rationale and would probably provoke similar expectations of healing.

The LSSM protocol was developed and tested over six months. First, the therapist was videotaped to establish the sequence of activities for subjects undergoing craniosacral and magnet therapy. A standardized sequence of CST was developed to permit a similarity of treatment among the CST subjects. The LSSM protocol was designed to mimic the CST protocol in terms of length of treatment sessions, sequence of interactions with the therapist, and overall treatment experience. During treatment, the subject lies supine on a table surrounded by small magnets, half of which are completely inactive. The active magnets are positioned such that they exert no field strength on the subject's skin or body. At each visit, the therapist confirms this by measuring the strength of the magnets with a Gaussmeter *[DC Magnetometer, Model 1, AlphaLab, Inc., 1280 South 300 West, Salt Lake City, UT 84101]*. Gaussmeter readings are designed to both improve the believability of the therapy and give the therapist something to do during the magnet sequence.

To further enhance active therapist involvement and simulate the CST maneuvers, additional inactive magnets contained within moveable pads are positioned on the subject in the same body areas treated in the CST protocol and moved to different sites at similar intervals.

The therapist then practiced the protocol every two weeks for 3 months to achieve a level of comfort with it that would approximate her comfort in administering CST. Although the therapist does not believe that magnet therapy will be effective, she is able to mask her doubts. In the videotaped encounters, we see her giving subjects copies of articles endorsing magnet therapy and we hear her explaining a plausible rationale for its efficacy.

A sham CST intervention was rejected as unfeasible. First, since the mechanism of CST is not completely known, it is unclear whether a sham procedure might exert a specific and unmeasurable therapeutic benefit [29]. Second, we suspected that an experienced CST practitioner would have difficulty in deviating from long established clinical skills and could not effectively perform sham maneuvers involving tissue palpation and release intended to parody CST. Furthermore, training an alternative therapist naïve to CST to perform a sham CST maneuver might be difficult and would introduce therapist variability into the randomization process [28].

### Outcome measures and study instruments

#### Primary outcome variables

The primary outcome variables assessed for this study include headache-specific quality-of-life, frequency of headache and self perceived benefit of the intervention (Table 3 describes measurement points for these variables). Detailed descriptions of these variables follow:

**Table 3:** Summary of Measures and Timing of Administration for Clinical Feasibility Trial of Craniosacral Therapy, 2006–2008

	**Week 0**	**Weeks 1 to 8**	**Weeks 8 to 16**	**Weeks 16 to 20**
**Variable or Instrument**	**Baseline Assessments**	**Baseline with Usual Care**	**Usual Care plus CST or LSSM**	**Post-treatment Assessments**

Demographic/Clinical Data	Week 0			
	ICHD* Diagnosis	Week 0		
Functional status – MOS SF-36	Week 0			Week 20
Expectation of benefit/Credibility †			(post 1^st^ treatment)	
Headache QOL – HIT-6 §	Week 0	Week 8		Week 16
Headache Diary: Frequency		Daily	Daily	
Perceived clinical change		Week 8		Week 16
Headache disability – MIDAS #	Week 0			Week 20
Headache Diary: Intensity and Duration		Daily	Daily	Daily
Headache Diary: Medication Use/Cost		Daily	Daily	Daily
Headache Diary: Health care visits		Daily	Daily	Daily
Satisfaction with care	Week 0	Week 8		Weeks 16, 20
Blood pressure and heart rate	Week 0		Weekly	
Therapist – Subject Interaction Analysis Videotaping Encounters**			Weeks 9,11, 13 & 15	

* International Classification of Headache Disorders, defined by expert members of the International Headache Society

† Based on a measure developed by Borkovec and Nau to measure credibility of psychological interventions [38,39].

§ Headache Impact Test was developed to measure headache-related quality of life [33].

# Migraine disability assessment score assesses the number of days of missed work or school in the past 3 months [36,37].

† Noldus 'Observer' Video-Pro System software facilitates the analysis of videotaped behaviors [42,43].

#### Headache Impact Test (HIT-6)

Headache-specific quality of life is measured as the change in headache-related disability at 8 weeks after randomization as assessed by the HIT-6. The HIT-6 is a validated questionnaire designed "to measure the impact headaches have on a person's ability to function on the job, at school, at home, and in social situations" [30]. It has good internal reliability [31]. The scale consists of six items that cover various content areas relevant to headache-related disability: pain, social functioning, role functioning, vitality, cognitive functioning, and psychological distress. A decrease of 2.3 HIT points (95% CI, 0.3 to 4.3) over six weeks among patients with chronic headache corresponds to a "somewhat better" self-reported rating on a global clinical change scale [31].

#### Headache frequency

**Figure 2:**
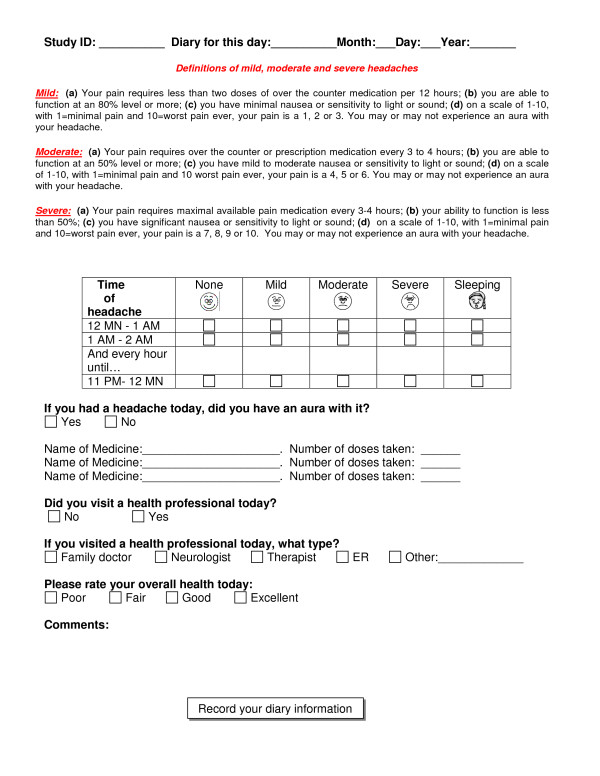
**On-line Daily Headache Diary of the Clinical Feasibility Trial of Craniosacral Therapy (CST), 2006–2008 Legend: Subjects had the option of filling out the diary on paper or on line.** Detailed instructions were provided at the time of enrollment.

the second primary outcome variable, is determined from the daily headache diary as the total number of headache hours during a 24-hour period. A second dimension of headache frequency is measured by the proportion of headache-free days experienced by a study participant (Figure 2). After receiving careful instruction, subjects record the presence and intensity of their headaches on an hourly basis. Subjects are also invited to comment on the nature of their headaches, the associated symptoms, and the suspected triggers.

#### Self-perceived benefit

of the intervention, one aspect of Specific Aim 3, is measured at the end of the 8-week treatment period. Subjects rate the perceived benefit of the therapy on a five point scale ranging from "greatly worsened my clinical condition" to "greatly improved my clinical condition". In addition, they assess the therapy as "more harmful than helpful", "neither harmful nor helpful", or "more helpful than harmful".

#### Secondary outcome variables

Secondary outcome variables include the following: headache intensity, headache-associated symptoms, medication used, cost of medication, health care utilization, change in general health status, study credibility, degree of headache related disability (MIDAS), and satisfaction with care. These are discussed below.

#### Headache intensity

As noted, the headache diary captures headache frequency and intensity, as well as other information. Intensity is defined as mild, moderate, or severe. Guides to grading headache intensity are included with each diary. The total hours of moderate or severe headache during a 24-hour period will serve as the measure of intensity. Subjects are invited to comment on the nature of their headaches, the associated symptoms, and the suspected triggers.

#### Headache-associated healthcare utilization

The diary also captures medication use and visits with a health care provider. (Figure 2).

#### Functional status and general health-related quality of life

The Medical Outcomes Study Short Form (SF-36) is a widely-used functional status instrument that assesses eight different health domains: physical functioning; bodily pain; role limitations due to physical problems; role limitations due to emotional problems; general mental health; social functioning; vitality; and general health perceptions [32]. A daily measure of well-being is included in the daily headache diary.

#### Headache-related disability: MIDAS

Disability, defined as the consequences of illness on ability to work and function, is measured using the migraine disability assessment score (MIDAS). Derived from the Headache Impact Test, MIDAS is a 7-item questionnaire that assesses the number of days during the previous three months that respondents missed work or school, experienced decreased productivity at work or home, or missed social engagements because of headaches. Test-retest reliability is acceptable, with Spearman's correlation coefficient ranging from 0.67 to 0.73. Cronbach's alpha is 0.83 [33,34].

#### Control variables

Control variables include socio-demographic and clinical characteristics, as well as evaluation of subject bias and protocol variability. These are described below.

#### Socio-demographic and clinical characteristics

Age, gender, ethnicity, religion, occupation, employment status, level of education, income, type of medical insurance, date of onset of migraine, history of use of CST, and presence of other medical conditions and diagnoses are collected at the time of the baseline interview. The medical history and physical exam performed by the PI are part of this data set. Subject weight, height, and vital signs are measured at the baseline visit.

### Evaluation of Subject Bias and Protocol Variability

#### Credibility and Expectation of Benefit

Subject credibility and expectations are assessed using an instrument adapted from a validated scale developed by Borkovec and Nau [35,36]. The instrument is administered just before the second visit. Data analysis will demonstrate whether subjects in the two groups have similar or different expectations of benefit. It has been shown that high expectations of treatment are closely related to better outcomes [37,38].

#### Quality control

Videotapes of the first, third, fifth, and seventh treatment sessions are used to provide feedback to the therapist to ensure standardization of the CST and LSSM treatment protocols as well as to identify any differences in therapist-subject behavioral interactions between the CST and LSSM treatments and thus control for interactional bias. Noldus 'Observer' Video-Pro System programmable software is used to code and analyze the selected videotaped sessions and quantitatively assess specific therapist and subject behaviors [39,40].

#### Adverse events

Reports of adverse events are obtained from subjects at each treatment session, from self reports recorded in the headache diaries or by direct contact with study staff. Adverse events are investigated by the study neurologist and are reported both to the UNC Institutional Review Board and the National Institutes of Health.

#### Analytic Strategy

Sample size and power calculations are based on the assumptions regarding the changes under treatment in two key measures of interest, namely, quality of life as measured by the Headache Impact Test (HIT) and headache frequency based on headache diary. Preliminary studies [41] have shown a mean HIT score of 63.7 with a standard deviation of 5.2 among migraine patients. Keeping the control values of HIT at this level, we estimate that with 45 subjects in each arm the study will have 80% power to detect a 3.5 points change in the HIT score. Based on our clinical experience we also estimate that, on average, migraine patients experience two headaches per week. With 45 patients per group, the study will have 80% power to detect a reduction in the headache frequency to one per week.

Regression modeling strategies will be used to test the primary hypothesis. A simple linear regression model with the HIT score at end of the study (16 weeks) controlling for HIT score at the baseline will be used to test for significant treatment effects. We will use repeated measures analysis on HIT measures to detect whether the change in HIT scores are significantly different between the two groups. Because of the correlated nature of the repeated measures, regression models using a generalized estimating equations (GEE) approach will be used to test the hypothesis of change. A significant time and treatment interaction will detect significant changes in the score. A similar analysis strategy using GEE methods will be used to analyze daily records of headache. All data analysis will be performed on an intent-to-treat basis. The GEE method assumes that missingness in data are completely at random. Under the missing at random assumption, it is possible to conduct analysis using multiple imputation procedures [42].

## Results

Recruitment into the study has proceeded as planned (Table 4). The majority of subjects are self-referred, having heard of the study either through email, flyers, or from a friend or family member. We have had mixed success in recruiting minorities for the study. Advertisements in the Spanish print media and in clinics serving minority populations were added to increase recruitment among the underserved. In addition, our Hispanic research assistant has helped us to recruit representative numbers of Hispanic subjects through personal contacts. Despite adding an African-American research assistant to the team, we have had less success recruiting African Americans.

**Table 4:** Recruitment in the Clinical Feasibility Trial of Craniosacral Therapy (CST) as of January, 2008

	**N**	**Percent**
Inquiries about study	226	
Never reached	45	20.0
Declined/deferred screening	12	7.1
Screened	169	74.9
Eligible, but declined	28	16.6
Ineligible	60	35.5
Headache characteristics	26	43.3
Psychiatric diagnoses	9	15.0
Hormonal issues	9	15.0
Other	16	26.7
Scheduled for consent	81	47.9
Referral pattern		
Health professionals*	28	16.6
Friends/family	28	16.6
Email advertisements†	46	27.2
Local print advertisements§	28	16.6
Flyers/brochures#	14	8.3
Recruitment letter**	6	3.6
UNC Website	1	0.6
Unknown	18	10.6

* Health professionals that referred patients to the study included neurologists, family physicians, and nurses.

† The university allows researchers to recruit subjects through an all-campus email message. The message goes out to students, faculty, and staff.

§ Print media included health-related publications and local English and Spanish-language newspapers

# Flyers and brochures were posted in physician's offices and clinics.

** A letter introducing the study was sent to 1000 adult patients who had been seen in the Family Medicine Center in the past 3 months.

At this time, 169 individuals have been screened for eligibility of which 109 were eligible to take part in the study. Five did not qualify during the baseline phase because of inadequate headache frequency. Nineteen have withdrawn from the study after giving consent. So far, seven subjects have failed to complete their headache diaries. Other subjects have dropped out due to intercurrent medical problems, accidents, or changes in conventional medication. No subject has dropped out due to an adverse event. So far, there has been equal drop out between groups after randomization.

Well over half of our subjects have adhered to completing a data-rich daily diary, either online or on paper, with minimal attrition. So far, we have lost 16% of subjects due to an inability to keep up with daily diary entry. Since the online diaries have been available, 86% have chosen to use them; 14% have chosen the paper diaries.

We have found that the online diary is superior to the paper diary for several reasons: (1) Missing data on the online diaries can be identified earlier and subjects can be prompted to remedy it. The paper diary is completed weekly and mailed in; it is not feasible to have subjects correct missing days once mailed to us. (2) Online diary data goes directly into a text file. Although there is still a potential for subject input error, this method eliminates the errors of secondary data entry. The time required for secondary data input is also eliminated. (3) Perhaps because they can type into it, subjects add more comments to the online diaries. We believe this provides a richer data set. (4) Finally, the online diaries are easier to copy. Several of our subjects have asked for a copy of the diary to share with their provider. Their individual diaries can be extracted from the online diary and sent to them at the end of their participation in the study.

## Discussion

Migraine is a common condition associated with significant disability [2]. Because pharmaceuticals for migraine provide incomplete relief of symptoms and are associated with significant side effects, many individuals seek complementary therapies, such as CST. Up to now, CST has not been studied rigorously. This report supports the feasibility of undertaking a rigorous randomized clinical trial of CST for migraine, using a standardized CST protocol and an innovative control protocol developed for this study.

In any study of complementary therapy in which there is the likelihood of a substantial placebo and expectation effect, a methodology is needed that permits detailed evaluation of the interaction between therapist and subject. We find that the Noldus system of interactional analysis using videotapes of the treatment sessions is a feasible, though time consuming, technique for achieving this goal. A detailed analysis of the videotapes will lead to refinements of the control (LSSM) protocol such that it more closely matches the intervention (CST) in terms of credibility and expectations of benefit. In addition, a qualitative evaluation of the post-intervention interview will yield important information about subject's satisfaction with the therapies.

We have established that it is possible to recruit enough subjects with migraine to obtain comparison groups with sufficient analytic power, though an enrollment period of 18 months would be needed to obtain at least 45 subjects in each group. Our data analyses, comparing CST with LSSM will allow us to determine effect sizes appropriate for a larger, definitive trial.

## Competing interests

The authors declare that they have no competing interests.

## Authors' contributions

Authors JDM, SAG, PC, KRF, and LW were involved in developing the original idea for funding. JDM was PI, and SAG, co-PI, on the funded proposal, with all other authors as co-applicants on the proposal. CS is providing oversight of data collection and statistical assessments. All authors participated in development of research protocols and design of the study. All authors read and approved the final manuscript.

## Pre-publication history

The pre-publication history for this paper can be accessed here:


